# Brain tumor surgery affecting the left frontal aslant tract and clinical outcome: a study of 23 glioma patients

**DOI:** 10.1016/j.bas.2026.106254

**Published:** 2026-08-04

**Authors:** Danial Nasiri, Alberto Consuegra, Corina Wyss, Lena Hostettler, Claire Descombes, Jonathan Wermelinger, Andreas Raabe, Philippe Schucht, Kathleen Seidel

**Affiliations:** a Department of Neurosurgery, Inselspital, Bern University Hospital, Switzerland; b Department of Neurology and Neurorehabilitation, Speech Therapy Section, Inselspital, Bern University Hospital, Switzerland

**Keywords:** Low grade glioma, Fiber tracking, Frontal aslant tract, Arcuate fasciculus, Penfield mapping, Language network

## Abstract

**Background:**

The frontal aslant tract (FAT) is associated with speech initiation, though its surgical relevance remains debated. The arcuate fasciculus (AF) is linked to phonological and semantic processing, with a well-established role for lasting outcomes.

**Research question:**

To investigate the impact of isolated and combined FAT and AF disruption on transient and lasting language deficits in glioma patients.

**Material and methods:**

We retrospectively analyzed 23 patients with non-contrast-enhancing IDH-mutant gliomas affecting the left FAT. Language was assessed pre-, intra-, and postoperatively. Awake speech mapping using Penfield stimulation was performed in 15 (65.2%) and neurophysiological monitoring under general anaesthesia in 8 patients. The FAT and AF were reconstructed by diffusion tensor imaging tractography, and tract integrity was classified as intact, partially disrupted, or fully disrupted. Postoperative and follow-up language deficits were correlated with the degree of tract disruption.

**Results:**

FAT disruption correlated with transient deficits (τ = 0.36, p = 0.059). AF involvement was associated with transient (τ= 0.30, p = 0.088) and persistent aphasia (τ = 0.53, p = 0.033). Combined FAT and AF damage was linked to an increased deficit of both immediate (τ = 0.44, p = 0.022) and lasting (τ = 0.49, p = 0.046) language outcomes.

**Discussion and conclusion:**

FAT injury contributes to transient aphasic symptoms, whereas AF disruption reflects persistent impairment. Concurrent FAT and AF involvement amplifies both short-term and lasting deficits, supporting a network-based approach to tract preservation in glioma surgery.

## Highlights

• Isolated FAT injury mainly caused transient speech deficits.
• AF disruption strongly predicted persistent aphasia.
• Combined FAT and AF damage worsened short- and long-term outcome.
• Network-based tract preservation is critical in glioma surgery.

## Abbreviations

AF: Arcuate Fasciculus
DTI: Diffusion-Tensor Imaging
FA: Fractional Anisotropy
FAT: Frontal Aslant Tract
fMRI: Functional Magnetic Resonance Imaging
GTR: Gross Total Resection
IOM: Intraoperative neurophysiological monitoring and mapping
NTR: Near Total Resection
ROI: Region of interest
SD: Standard Deviation
SFG: Superior Frontal Gyrus
SMA: Supplementary Motor Area
STR: Subtotal Resection

## 1 Introduction

Preservation of language function during glioma resection relies on the integrity of subcortical white matter pathways. Among available non-invasive methods, diffusion tensor imaging (DTI) has proved particularly valuable for preoperative visualization of these fiber tracts, enabling neurosurgeons to assess potential tract infiltration and plan resections that minimize postoperative language deficits (Abdullah et al., 2013; Salvati et al., 2021; Ille et al., 2018; Chang et al., 2015).

The frontal aslant tract (FAT) is a white matter pathway that predominantly originates from the convexity of the superior frontal gyrus, with additional contributions from medial frontal regions including the supplementary motor area (SMA), and projects to the inferior frontal gyrus (IFG). It has been implicated in speech initiation and verbal fluency (Catani et al., 2013; La Corte et al., 2021; Aliaga-Arias et al., 2024; Chernoff et al., 2019; Burkhardt et al., 2022; Dick et al., 2019; Tagliaferri et al., 2024). Yet, its relevance in surgical planning remains debated. Some advocate for its preservation to reduce the risk of permanent language impairments, while others consider its involvement to result primarily in transient speech initiation deficits akin to SMA syndrome (La Corte et al., 2021; Young et al., 2020, 2022; Agyemang et al., 2023; Briggs et al., 2021). Considering the onco-functional balance, this would raise question if those transient deficits might be tolerated in the sake of a more radical tumor resection.

The arcuate fasciculus (AF) is a prominent tract within the language network, contributing to both the dorsal phonological and ventral semantic stream supporting functions beyond straightforward speech repetition (Bernal and Ardila, 2009; Catani and Mesulam, 2008; Fridriksson et al., 2013; Giampiccolo and Duffau, 2022; Ivanova et al., 2021). Evidence indicates that injury to major language-related white matter pathways, such as the AF, during glioma surgery results in permanent aphasia, underscoring its clinical importance (Giampiccolo and Duffau, 2022; Tuncer et al., 2021; Forkel et al., 2014).

**Fig. 1:**
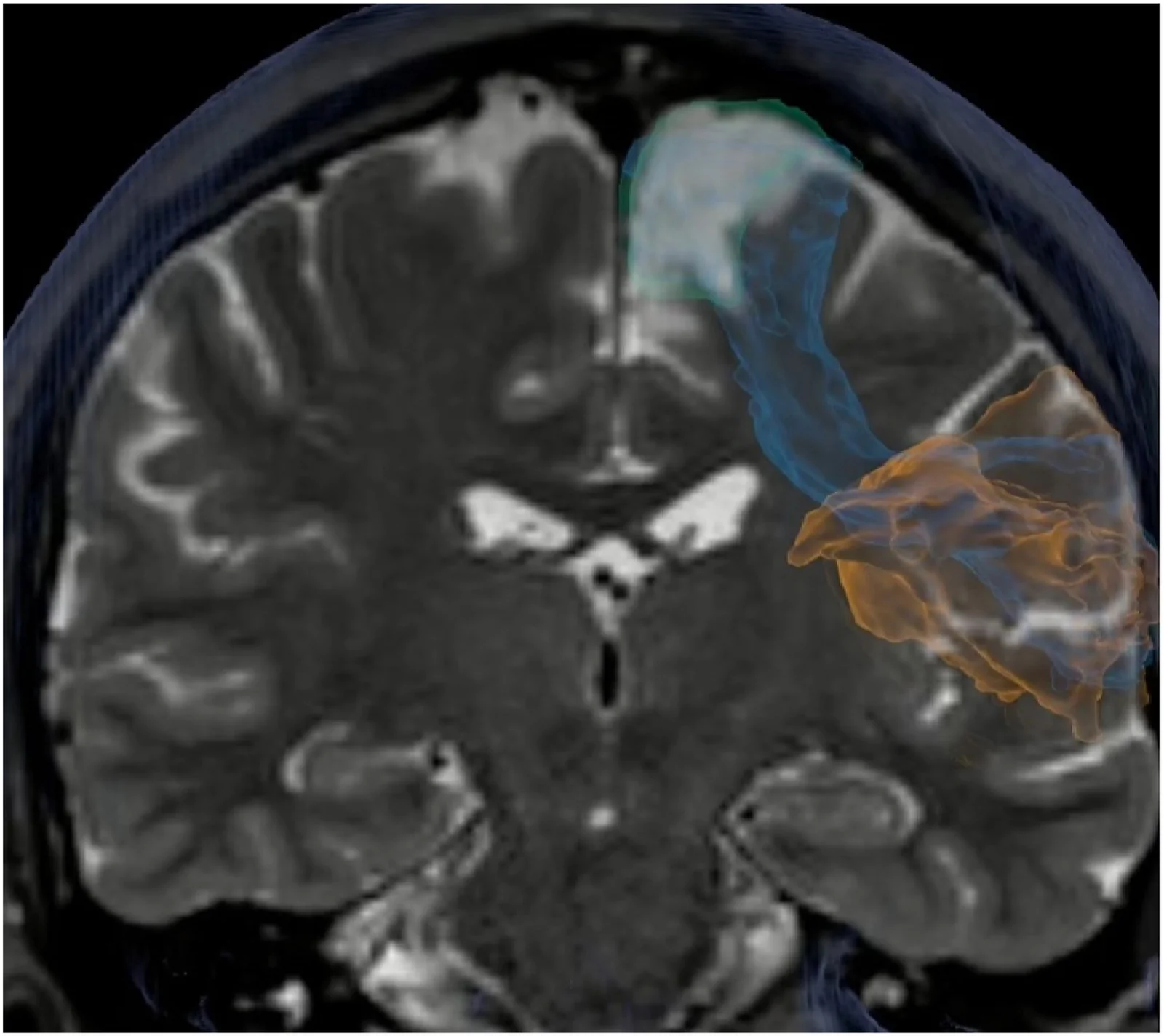
Coronal slide of a T2 postoperative MRI. The resection cavity of the low-grade glioma involving the supplementary motor area region of the superior frontal gyrus is depicted in green. The frontal aslant tract crossing the glioma is visualized in blue, whereas the arcuate fasciculus is shown in orange. DTI fiber-tracking parameters were set to FA = 0.15, length = 50 mm, angulation = 45° as described under methods 2.5.

Against this backdrop, the present study examines the functional significance of the FAT integrity alone or combined with the AF integrity in patients who underwent resection of non-contrast-enhancing gliomas in the left frontal lobe (Fig. 1). We investigate whether disruption of these tracts is associated with transient and lasting language deficits. Further, we investigated the assumption that FAT disruption invariably corresponds to a transient, reversible SMA-related syndrome.

## 2 Material and methods

### 2.1 Ethical considerations

We conducted a retrospective, single-center study, approved by the Bernese Cantonal Ethics Commission (Project ID, 2024-02510). All patients provided general consent, and the study followed STROBE guidelines.

### 2.2 Study population

We screened 286 patients and included 23 patients who underwent surgery between January 2019 and December 2024 (see Supplementary Figure 1). The inclusion criteria were IDH-mutant gliomas without contrast enhancement located in the left frontal lobe affecting the FAT (Louis et al., 2021). Exclusion criteria included glioblastomas, contrast-enhancing gliomas, right hemisphere tumors, and unavailable DTI tractography. This study deliberately focused on predominantly non-contrast-enhancing, IDH-mutant gliomas, as their general longer survival enables longitudinal evaluation of postoperative language recovery. The speech-eloquent hemisphere was identified using fMRI (7/23, 30.4%) or neuropsychological testing (22/23, 95.7%).

### 2.3 Language assessment

Language assessments were performed preoperatively (median 7.5 days before surgery), intraoperatively, postoperatively (median 2 days after surgery), and at follow-up (median 64 days after surgery). Preoperative tests evaluated spontaneous speech, auditory comprehension, object naming, and writing. Intraoperative monitoring included spontaneous speech and a picture-naming task during cortical and subcortical mapping. Only items successfully named preoperatively were used. Postoperative assessments included informal bedside screening, and follow-up assessments repeated the preoperative test battery.

**Fig. 2:**
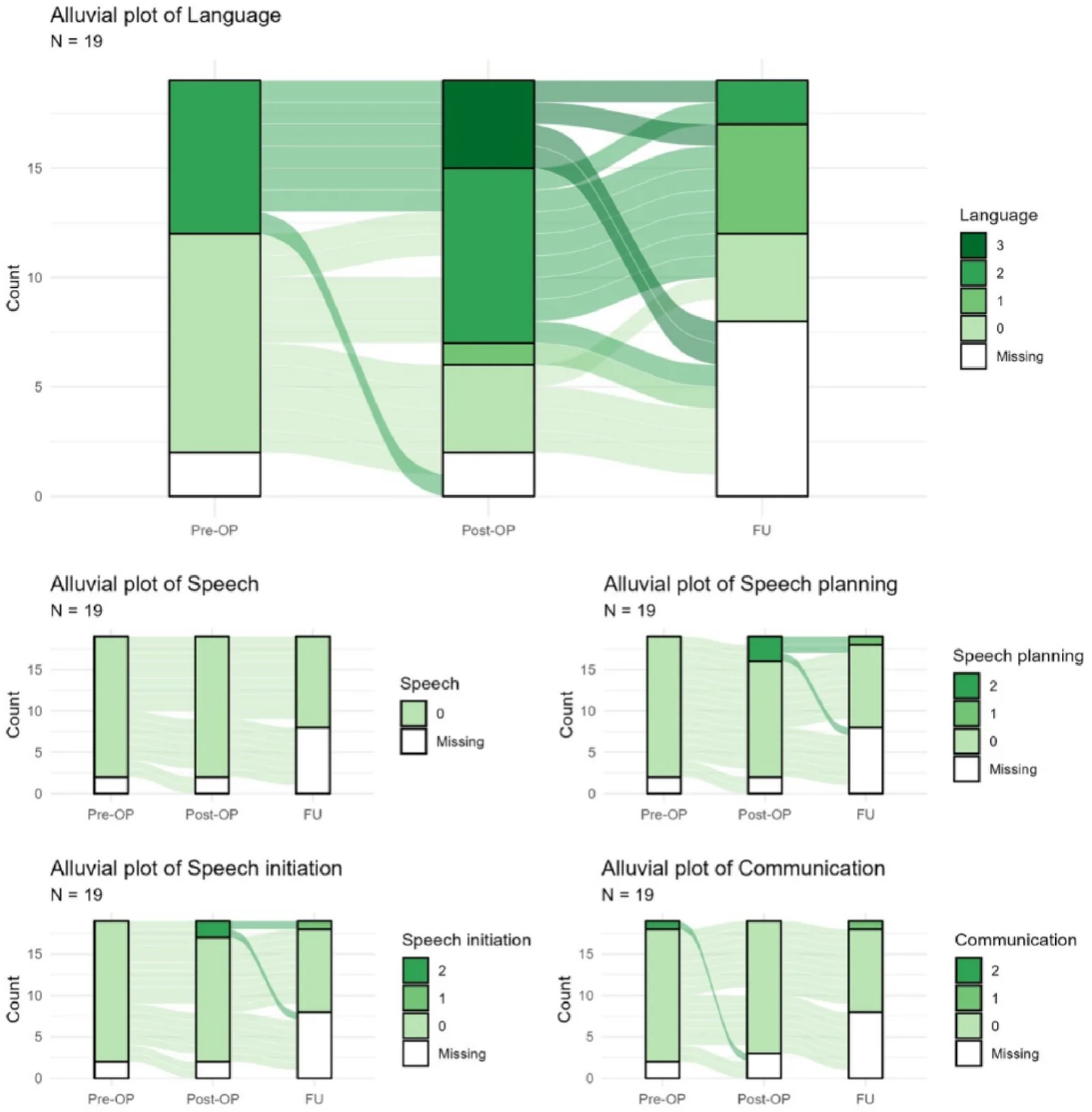
Illustration of the evolution of the distribution of deficits across five linguistic deficit categories at different time points. Scores range from 0 (no deficit) to 3 (severe deficit), with intermediate grades indicating mild and moderate impairment. The five linguistic deficit categories are defined as follows: language = aphasia as a language disorder, speech = dysarthria as a speech disorder, speech planning = apraxia of speech as a speech planning disorder, speech initiation = difficulties in producing speech, communication = pattern of cognitive communication disorder.

**Fig. 3:**
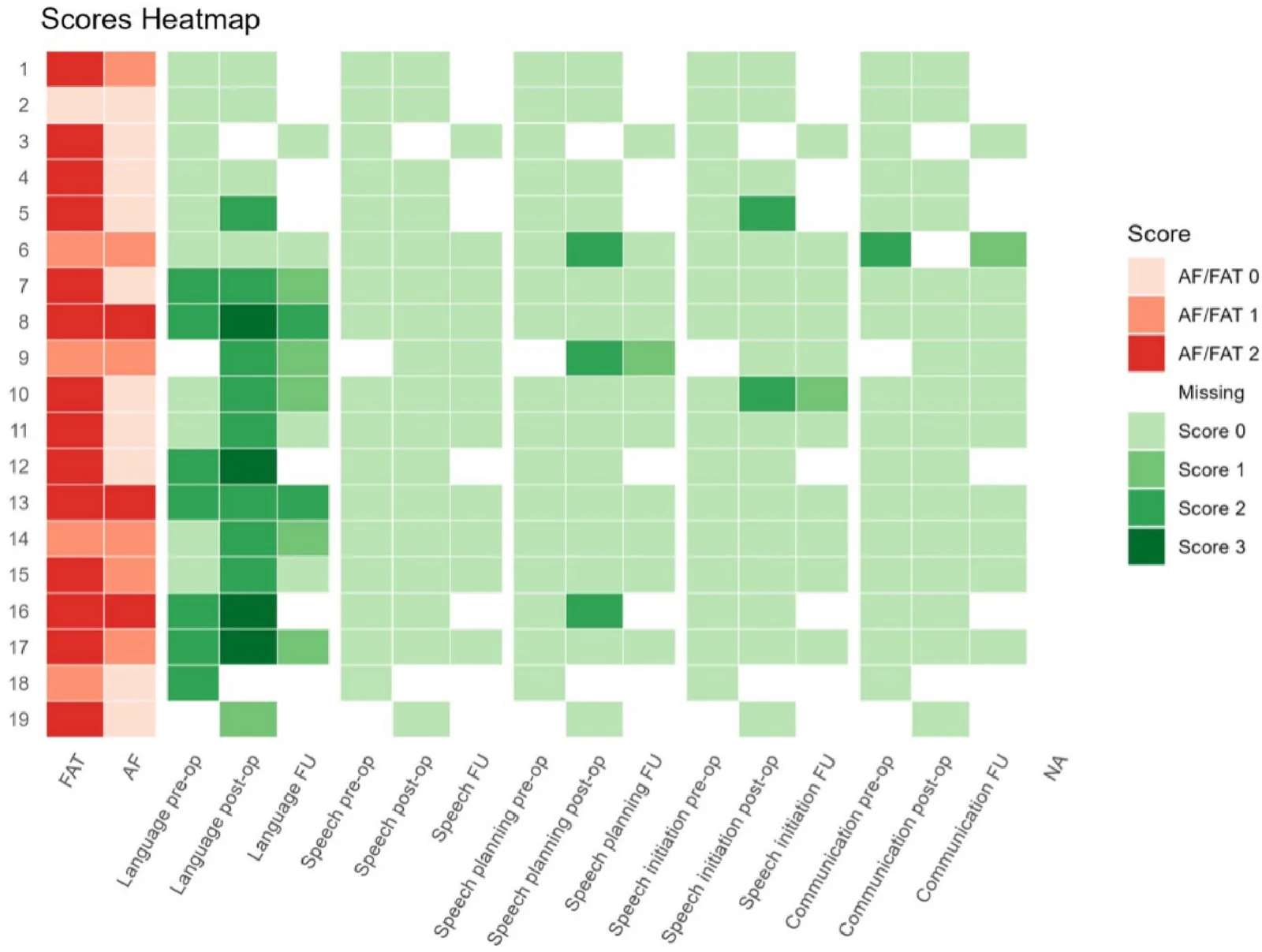
Heatmap illustrating disruption of the FAT and AF (red, graded 0 = intact, 1 = partially disrupted, 2 = completely disrupted), and the deficits across five linguistic categories at preoperative, immediate postoperative and 3 months follow-up assessments (green). The five linguistic deficit categories are defined as follows: language = aphasia as a language disorder, speech = dysarthria as a speech disorder, speech planning = apraxia of speech as a speech planning disorder, speech initiation = difficulties in producing speech, communication = pattern of cognitive communication disorder. The shades of the colors indicate the severity of linguistic impairment with darker colors indicating more severe deficits. The illustrated data is from 19 patients, due to 4 patients with missing values for language assessment.

Five linguistic deficit categories were defined: "language," "speech," "speech planning," "speech initiation," and "communication," based on speech-language disorder literature (Jordan and Hillis, 2006; McSween et al., 2024; Togher et al., 2023). These categories consisted of aphasia as a “language” disorder, dysarthria as a “speech” disorder and apraxia of speech as a “speech planning” disorder. Further, “communication” was defined as a cognitive communication disorder. In addition, some patients had difficulties in producing speech on their own and, in particular, in initiating utterances. The category “speech initiation” was defined for such deficits. (Fig. 2, Fig. 3).

All language assessments were performed by two certified speech and language therapists. The categorization of linguistic deficits was based on established speech-language pathology literature. However, due to the different severity of language deficits and socioeconomic background of the individual patients, no universally accepted defined classification system for postoperative language deficits exists in this context. Thus, the categories were defined pragmatically according to the limited available evidence and expert clinical judgment.

### 2.4 Intraoperative neurophysiological mapping protocol

In the patients which were operated in an awake surgery setting, Penfield stimulation for mapping of language function was performed with an ISIS IOM System (Inomed®, Emmendingen, Germany) (Seidel et al., 2024). We applied square-wave biphasic current with a pulse width (PW) of 0.6 ms and a frequency of 50 Hz, using a bipolar stimulation probe with an interpolar distance of 8 mm and tip diameters of 2 mm (Inomed® REF 522 624). The stimulation current intensity was determined over the primary motor area of the tongue while the patient was counting, to check for dysarthria. This stimulation intensity, between 3 and 6 mA, was then used for further mapping of the speech eloquent cortex. During the cortical mapping, electrocorticography was recorded to detect after-discharges or spike activity with the help of subdural spider electrodes (AdTech® REF VG04A-IS00X-0KG).

For those patients undergoing intraoperative neurophysiological monitoring and mapping (IOM) under general anaesthesia, the detailed methods for motor evoked potential monitoring and continuous subcortical dynamic mapping with the monopolar suction probe are described in our previous publication (Seidel et al., 2025). The choice for awake surgery or resection under IOM was done accordingly to tumor location and patient cooperation.

### 2.5 Measurement of the FAT and AF

FAT and AF were reconstructed using BrainLab software (BrainLab, Munich, Germany) on pre- or postoperative DTI datasets. For FAT, the superior frontal gyrus was selected as the region of interest, connected to Broca's area. Fiber tracking parameters included FA threshold 0.15, minimum fiber length 50 mm, and maximum angulation 45°. For AF, a predefined template was used with the same parameters. (Tagliaferri et al., 2024). To assess the robustness of tract reconstruction, additional analyses were performed using different combinations of minimum fiber length, fractional anisotropy, and angulation thresholds. The 50-mm minimum fiber length provided the most anatomically plausible and reproducible reconstructions and was therefore used for the final analyses.

### 2.6 Classification of speech and language deficits and tract integrity

Deficits were classified into five linguistic deficit categories (see 2.3): language, speech, speech planning, speech initiation, and communication. Each was scored for severity on a four-point ordinal scale (0–3; no to severe deficit). To assess tract integrity, preoperative DTI reconstructions of the FAT and AF were fused with postoperative T2-weighted images. As postoperative DTI datasets were unavailable in the majority of cases, preoperative DTI datasets were fused with both pre- and postoperative T2-weighted imaging to evaluate whether the tumor and surgical resection cavity overlapped with or disrupted the preoperatively reconstructed FAT and AF. Tract disruption was classified as "intact" (score 0), "partial disruption" (score 1) or "total disruption" (score 2). This allowed comparison of functional deficits across domains with the structural integrity of the FAT and AF at various time points.

### 2.7 Statistics

For inferential statistics, we evaluated the association between each pair of ordinal scores by computing Kendall's tau correlation coefficients. Based on prior evidence and our clinical hypothesis, greater involvement of the FAT and AF was expected to be associated with more severe postoperative language deficits rather than improved language performance. We therefore tested for positive associations between tract involvement and language deficit severity using one-sided hypothesis tests. Thus, all correlation coefficients were tested using a one-sided test against the null hypothesis of no correlation. The significance level for p-values was set at 0.05. The statistical analyses were performed using R (R 4.5.0 and 4.6.1, R Core Team (2025-2026)) and RStudio (2024.12.1.563 and 2026.5.0.218, Posit team (2025-2026)).

## 3 Results

### 3.1 Patient characteristics

The cohort included 23 patients (7 women, 16 men; median age 48.0 years). Of the patients, 87.0% were right-handed, and 13.0% were left-handed. In those 3 left-handed patients, the dominance of the left hemisphere was confirmed with functional MRI and neuropsychological testing, while the third patient had solely neuropsychological testing. Surgery was performed using an asleep-awake-asleep protocol in 15 patients (65.2%) and under general anaesthesia with IOM in 8 patients (34.8%). In one of those 8 patients an AF involvement was suspected preoperatively, however an awake procedure was not feasible due to severe preoperative language deficits. In all other cases with presumed AF affection, surgery was performed using an asleep–awake–asleep protocol. Tumor diagnoses included oligodendroglioma CNS WHO grade 2 (39.1%) and grade 3 (17.4%), astrocytoma IDH-mutant CNS WHO grade 2 (17.4%) and 3 (21.7%), and CNS WHO grade 4 astrocytoma in 1 patient (4.4%). Gross total resection was achieved in 47.8% of patients and near total resection in 13.0% and subtotal resection in 39.1%.

See Supplementary Table 1 for baseline characteristics.

### 3.2 Distribution of speech and language deficits

For the definition of the linguistic deficit categories see method section 2.3, 2.6. Postoperative correlation analyses were based on 17 patients with complete language assessment data. At the 3-month follow-up, 11 patients had complete language assessment data for correlation analyses, whereas overall follow-up data were available for 19 patients.

#### 3.2.1 Preoperative assessment

7 of 23 patients (30.4%) had a language deficit. No speech, speech planning, or initiation deficits were observed. Communication deficits were present in 1 patient (4.3%).

#### 3.2.2 Postoperative assessment

Language deficits were present in 13 patients (56.5%), corresponding to 10 patients (43.5%) with a new or worsened language deficit. No speech deficits were observed. Speech planning deficits occurred in 3 patients (13.0%), initiation deficits in 2 patients (8.7%), and communication deficits in 1 patient (4.3%).

#### 3.2.3 3-Month follow-up

Language deficits were present in 7 patients (30.4%). 9 of the 10 patients with new or worsened postoperative language deficits had improved by follow-up, while follow-up data were unavailable for 1 patient. No speech deficits were observed. Speech planning deficits persisted in 1 patient (4.3%), initiation deficits in 1 patient (4.3%), and communication deficits remained present in 1 patient (4.3%).

Fig. 2, Fig. 3 show the distribution of the five linguistic deficit categories across the preoperative, postoperative, and follow-up assessments.

### 3.3 Immediate postoperative language deficits and association with FAT and AF integrity

FAT integrity showed a significant correlation with immediate postoperative language outcomes (Kendall τ= 0.36, p = 0.059). Patients with higher FAT disruption exhibited more severe postoperative language deficits (see Fig. 3). AF integrity showed a moderate, non-significant correlation with immediate postoperative deficits (Kendall τ = 0.30, p = 0.088).

### 3.4 Persistent deficits and association with FAT and AF integrity

**Fig. 4:**
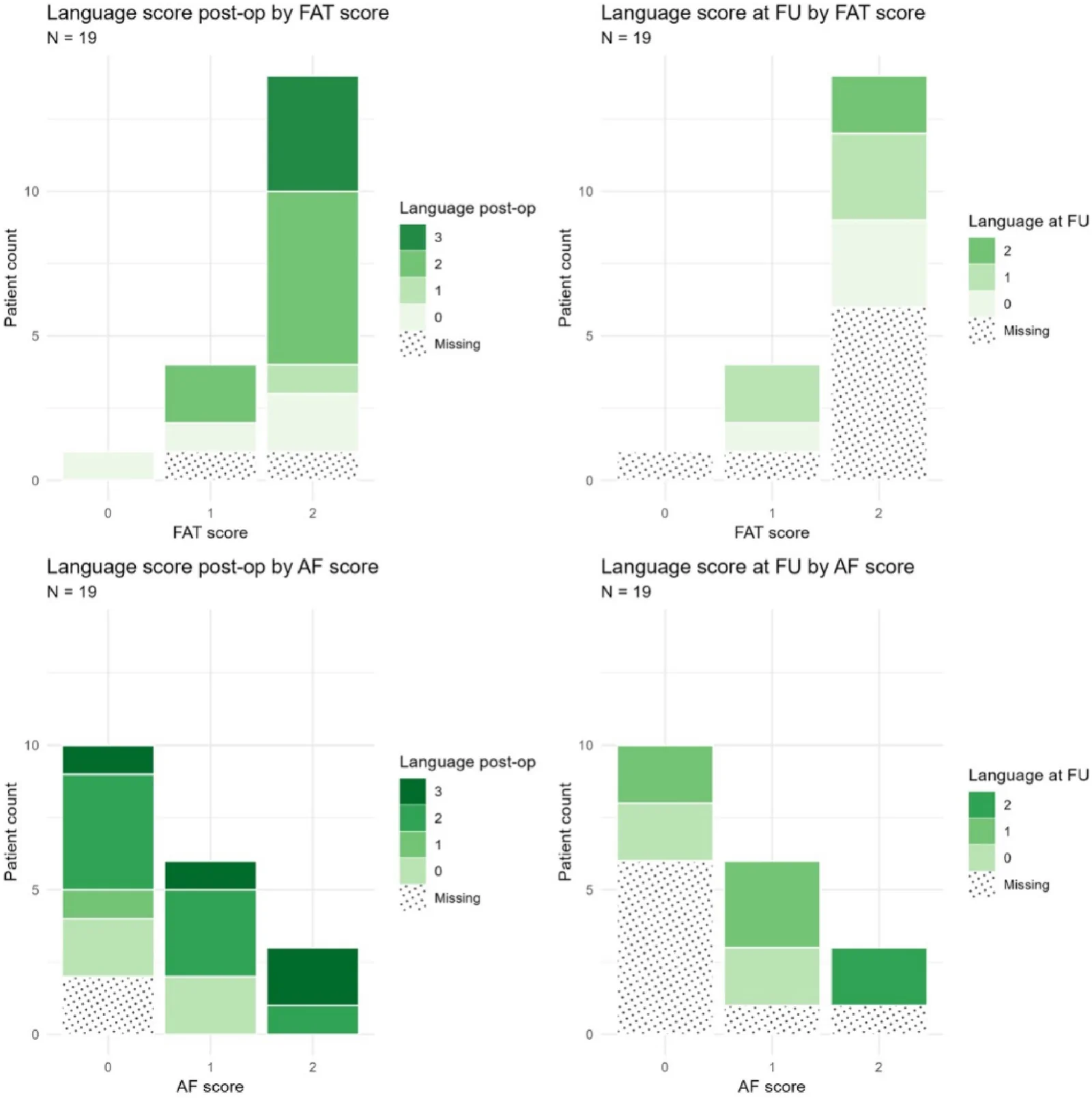
Distribution of postoperative (left) and follow-up (right) language deficits for the different levels of FAT (above) and AF (below) disruption. The x-axis denotes tract integrity (0 = intact, 1 = partially disrupted, 2 = completely disrupted), while the y-axis indicates the (absolute) affected patient number. FAT/AF score stands for the tract disruption, while 0 is no and 2 complete disruptions in the DTI as described in the method section 2.6. A language deficit was defined as aphasia as described in the method section 2.3 while the darkest color stands for the most severe deficit as described in the method section.

Persistent deficits at follow-up were significantly associated with AF integrity (Kendall τ = 0.53, p = 0.033). Complete disruption of AF was most strongly linked to lasting language dysfunction (see Fig. 4). FAT integrity showed a weak, non-significant association with persistent deficits (Kendall τ = 0.10, p = 0.371).

### 3.5 Association of deficits with FAT and AF integrity combined

The summed FAT and AF disruption scores showed a moderate, significant correlation with immediate postoperative deficits (Kendall τ = 0.44, p = 0.022) and lasting deficits (Kendall τ = 0.49, p = 0.046) (Supplementary Table 2).

## 4 Discussion

In this retrospective cohort of 23 patients undergoing resection of non-contrast-enhancing IDH-mutant gliomas of the left frontal lobe, we observed distinct patterns of postoperative language impairment associated with involvement of the FAT and AF. Involvement of the FAT was predominantly associated with transient postoperative deficits, whereas persistent impairments were more frequently observed in patients with AF involvement. Patients with combined FAT and AF involvement showed the poorest recovery. Although these findings should be interpreted cautiously given the sample size and study design, they support the concept that different white matter pathways may contribute differently to postoperative language recovery.

### 4.1 FAT impairment – SMA syndrome?

The FAT has been shown to be involved in speech initiation and fluency (Catani et al., 2013; Catani and Mesulam, 2008). Lesion and stimulation studies have demonstrated that FAT disruption can cause transient speech arrest or initiation delays, often resolving over days to weeks (Ille et al., 2018; Young et al., 2020, 2022; Fujii et al., 2015). This evidence underpins the first school of thought, which situates the FAT within the SMA initiation network and predicts reversible deficits.

Our findings indicate that isolated FAT lesions were associated with postoperative language impairments, including initiation deficits and aphasic symptoms (Fig. 2, Fig. 3, Fig. 4). While these deficits were common immediately after surgery, they were predominantly transient and largely resolved at follow-up. This observation is consistent with previous reports showing that FAT injury can be associated with language disturbances while rarely resulting in permanent aphasia (Catani et al., 2013; La Corte et al., 2021; Vassal et al., 2014; Kinoshita et al., 2015; Chernoff et al., 2018; Zhong et al., 2022).

The transient nature of these deficits may reflect functional recovery and network reorganization, but it is also compatible with the emerging view that the FAT supports domain-general executive control processes that contribute to language in the dominant hemisphere rather than serving an exclusively language-specific function. Consistent with this perspective, recent studies have implicated the FAT in broader cognitive and action control mechanisms beyond language (Dick et al., 2019; Serrano-Sponton et al., 2024; Tagliaferri et al., 2023). Given the limited sample size and the absence of non-language control measures in the present study, our findings should therefore be interpreted as supporting an association between FAT lesions and transient postoperative language deficits, rather than establishing a language-specific role of the FAT.

### 4.2 AF and persistent language impairment

By contrast, the AF is a well-established component of the dorsal stream as conceptualized by Hickok and Poeppel's dual-stream model (Hickok and Poeppel, 2007, 2015). It provides the principal perisylvian link between posterior temporal regions and frontal language cortices (Catani and Mesulam, 2008; Catani et al., 2005). Consistent with prior studies, we found that AF disruption was significantly associated with (short-term and) persistent aphasia at follow-up (Salvati et al., 2021; Tuncer et al., 2021). This is in line with evidence that posterior AF segments, particularly at the superior temporal gyrus and perisylvian junction, are especially critical for lasting language function (Tuncer et al., 2021; Saur et al., 2008). Several studies suggest that these two regions within the AF are particularly vulnerable to persistent language deficits, likely due to the dense convergence of subcortical white matter pathways in these areas (Catani and Mesulam, 2008; Tuncer et al., 2021; Forkel et al., 2014; Saur et al., 2008).

### 4.3 Combined FAT and AF disruption

Assessing both tracts concurrently allowed us to observe that combined FAT and AF involvement was associated with higher rates of postoperative aphasia and reduced recovery. While we did not focus in this study on cases of isolated AF injury to directly contrast, our findings suggest that combined tract damage may amplify the emergence of both transient and persistent deficits. This observation contributes new evidence to the debate on tract-specific versus network-level vulnerability of language function in glioma surgery (Duffau et al., 2014; Nieberlein et al., 2023).

### 4.4 Clinical implications

From a surgical perspective, our findings support careful consideration of both the FAT and AF during preoperative planning and intraoperative mapping. Preservation of the FAT may contribute to reducing transient postoperative language disturbances, whereas preservation of the AF remains particularly important for lasting language outcome (Vooijs et al., 2024). Sparing the AF, particularly its posterior segments, remains paramount for maintaining (short- and) lasting language abilities. While intraoperative mapping remains the gold standard for functional preservation in language-eloquent surgery, additional advanced tractography provides a practical framework to identify these pathways and to guide resections that maximize tumor removal while safeguarding language function (Salvati et al., 2021; Ille et al., 2018; Tuncer et al., 2021; Seidel et al., 2024; Fujii et al., 2015; Intraoperative mapping and monitoring during, 2022). Studies have shown that the potential for language reorganization in glioma patients is strongly region-dependent: frontal language areas such as the left inferior frontal gyrus often exhibit high plasticity with perilesional or distributed intrahemispheric reorganization, whereas regions with stronger dependence on critical subcortical pathways - such as the ventral premotor cortex, the insula (particularly when opercular regions are also involved), and parts of the temporal lobe connected via the arcuate and superior longitudinal fasciculi - show more limited plasticity and frequently retain essential language function within or near the tumor, thereby constraining surgical resection (Nieberlein et al., 2023; Leonard et al., 2024; Herbet and Duffau, 2020). Those findings align with the results of our series in which patients with a combined lesion of the FAT and AF show a low potential for recovery.

### 4.5 Limitations

Study limitations are its retrospective design, including missing data and variability in DTI acquisition. Most patients underwent either pre- or postoperative DTI, limiting cohort consistency. The small sample size, although comparable to other studies (Young et al., 2020; Tuncer et al., 2021; Kinoshita et al., 2015; Herbet and Duffau, 2020), restricts generalizability. Results are exploratory and should be interpreted cautiously.

Another limitation is the use of diffusion MRI tractography to assess white matter tract involvement. Although tractography is widely used for preoperative planning and anatomical visualization, its accuracy may be affected by tumor-related changes such as infiltration, edema, mass effect, tract displacement, and crossing fibers. Consequently, reconstructed fiber tracts represent an approximation of the underlying anatomy rather than a direct measure of white matter integrity. In addition, postoperative tract involvement was inferred by combining preoperative tractography with postoperative structural MRI, which may be affected by brain shift, postoperative edema, cavity collapse, blood products, and other surgery-related distortions, potentially altering the spatial relationship between the resection cavity and the reconstructed fiber tracts. Therefore, the observed associations between tract involvement and postoperative language deficits should be interpreted with appropriate caution. Longitudinal postoperative diffusion MRI acquired after recovery from acute postoperative changes would provide a more reliable assessment of tract integrity but was not routinely available in this retrospective cohort.

A further limitation is that tract involvement was determined using a clinically established deterministic tractography workflow rather than a hypothesis-independent disconnectome approach, which may have limited the detection of alternative disconnection patterns despite reflecting current neurosurgical practice.

Right hemispheric lesions were excluded, although they may contribute to FAT involvement. The focus on left-sided lesions limits generalizability, but bilateral involvement will be explored in future studies. Additionally, we did not analyze other relevant tracts such as the inferior fronto-occipital fasciculus (Giampiccolo and Duffau, 2022).

Further, speech assessments were individualized, limiting standardization and interindividual comparisons. Future studies will address higher neuropsychological functions like processing speed. The absence of standardized postoperative speech-language pathology evaluations limited the ability to retrospectively distinguish between established diagnostic entities, necessitating a descriptive classification of observed speech and language impairments.

Preoperative language deficits were not correlated with FAT integrity due to a small sample. Although preoperative and lasting deficits overlapped, tumor invasion of white matter tracts, particularly the FAT, likely accounts for deficits, though surgical contributions must also be considered. The increased incidence of immediate postoperative deficits suggests surgical disruption of white matter pathways. The higher incidence of immediate postoperative deficits suggests that surgical manipulation of white matter pathways contributes to these impairments, but this interpretation requires confirmation in larger prospective studies.

Tumor heterogeneity, particularly with respect to lesion size, represents an additional limitation of this study. Variability in tumor volume may have influenced both the extent of resection and the degree of white matter involvement, thereby potentially affecting functional outcomes and limiting the comparability of cases within the cohort.

## 5 Conclusion

In summary, our data integrate and extend the two prevailing schools of thought on the FAT. We found that FAT disruption is not limited to transient speech initiation deficits but can cause clinically relevant, transient aphasia. The predominantly transient nature of these deficits is consistent with functional recovery and the emerging view that the FAT contributes to domain-general executive control processes supporting language rather than serving an exclusively language-specific function. The novel observation that concurrent FAT and AF involvement confers especially poor language outcomes further highlights the importance of a network-based view of language pathways in neurosurgical planning and patient counselling.

## Ethical approval

The study was approved by the Bernese local Ethics Committee (Project ID, 2024-02510). All patients provided informed consent for treatment and inclusion of their anonymized data in research analyses.

## Author contributions

The study conception and design were elaborated by D.N., A.C., and K.S. Material preparation and data collection were conducted by D.N., A.C., C.W. and L.H. Data analysis was performed by D.N., C.D., J.W. and K.S. The first draft of the manuscript was written by Danial Nasiri and all authors commented on previous versions of the manuscript. All authors read and approved the final manuscript.

## Funding

Open access funding provided by 10.13039/100009068University of Bern. The Department of Neurosurgery funded the study.

## Declaration of interests

The authors declare that they have no known competing financial interests or personal relationships that could have appeared to influence the work reported in this paper.
