# Supplementary material for: Brain tumor surgery affecting the left frontal aslant tract and clinical outcome: a study of 23 glioma patients
Source: Brain Spine. 2026 Aug 4;6:106254. doi: 10.1016/j.bas.2026.106254 (PMC13499198; doi:10.1016/j.bas.2026.106254)
Supplement: Supplementary file 1 [file mmc1.docx]

| Patient no° | Age | Sex | DTI preop | DTI postop | FAT preop (in cc) | FAT postop (in cc) | Histology | Handedness | Anaesthesia |
| --- | --- | --- | --- | --- | --- | --- | --- | --- | --- |
| 1 | 42 | F | Y | N | 23.8 | na | Astrocytoma, 2 | Right | IOM-GA |
| 2 | 31 | M | Y | N | 64.9 | na | Astrocytoma, 2 | Right | Awake |
| 3 | 50 | M | Y | N | 56.1 | na | Oligodendroglioma, 2 | Right | IOM-GA |
| 4 | 53 | M | Y | N | na | 18.1 | Astrocytoma, 3 | Right | IOM-GA |
| 5 | 53 | M | Y | N | 32.3 | na | Astrocytoma, 3 | Right | Awake |
| 6 | 39 | M | Y | N | 22.3 | na | Astrocytoma, 3 | Left | Awake |
| 7 | 45 | M | Y | Y | na | 26.7 | Astrocytoma, 3 | Left | IOM-GA |
| 8 | 50 | M | Y | N | 24.0 | na | Astrocytoma, 3 | Right | Awake |
| 9 | 59 | M | Y | Y | 37.2 | 14.1 | Oligodendroglioma, 2 | Right | IOM-GA |
| 10 | 50 | M | Y | Y | 21.4 | 17.6 | Astrocytoma, 2 | Left | Awake |
| 11 | 47 | M | Y | Y | 35.6 | na | Oligodendroglioma, 2 | Right | Awake |
| 12 | 55 | F | Y | Y | 4.77 | 0.78 | Astrocytoma, 4 | Right | Awake |
| 13 | 25 | M | Y | N | na | na | Oligodendroglioma, 2 | Right | Awake |
| 14 | 41 | M | Y | N | 34.9 | na | Oligodendroglioma, 2 | Right | Awake |
| 15 | 33 | F | Y | N | 50.6 | na | Oligodendroglioma, 2 | Right | Awake |
| 16 | 42 | F | Y | N | 30.1 | na | Oligodendroglioma, 2 | Right | Awake |
| 17 | 62 | M | Y | N | 47.7 | na | Oligodendroglioma, 3 | Right | IOM-GA |
| 18 | 38 | M | Y | N | 14.0 | na | Oligodendroglioma, 3 | Right | Awake |
| 19 | 64 | F | Y | Y | 83.6 | na | Oligodendroglioma, 3 | Right | Awake |
| 20 | 71 | M | Y | N | 102.0 | / | Oligodendroglioma, 3 | Right | IOM-GA |
| 21 | 65 | M | Y | N | 39.9 | / | Oligodendroglioma, 3 | Right | IOM-GA |
| 22 | 47 | F | Y | N | 17.2 | / | Astrocytoma, 2 | Right | IOM-GA |
| 23 | 52 | F | Y | Y | 21.4 | / | Oligodendroglioma, 2 | Right | Awake |

Supplementary Table 1: Patient demographics. DTI = diffusion-tensor imaging, F = female, FAT = frontal aslant tract, IOM-GA = intraoperative neurophysiological monitoring-general anaesthesia, na = postoperative DTI was not available, M = male, N = no, Y = yes. For more information on hemispheric dominance see section 3.1.

Supplementary Table 2: Correlation analyses for language outcome based on FAT and AF integrity.

Supplementary Figure 1: Flow chart illustrating the inclusion and exclusion process of 286 screened patients.
